# Medical Wards of the Mercy Hospital

**Published:** 1863

**Authors:** N. S. Davis

**Affiliations:** Professor of Practical and Clinical Medicine, &c.


### MEDICAL WARDS OF THE MERCY HOSPITAL.

### ERYSIPELAS,—SULPHITE OF LIME, &c.

BY N. S. DAVIS, M.D., Professor of Practical and Clinical Medicine, &c.

During the last half of March and the first part of April, 1863, erysipelas was more prevalent in this city than usual; and, as a consequence, several very severe cases, occurring among the poorer classes, were received into the Mercy Hospital. They constituted the subject of several clinical lectures to the class attending the summer course of instruction in the Chicago Medical College, (Medical Department of Lind University,) thereby enabling the students to study the disease practically in all its stages. One item in the treatment of some of these cases, has induced us to report them somewhat in detail:—

CASE I.—Mr. M., was admitted into the medical ward of the Mercy Hospital, April 2d. He had been sick five or six days previously. At the time of admission a well-marked erysipelatous inflammation occupied the whole face, (except the chin,) the ears, the mastoid spaces, and part of the scalp. The tumefaction was so great as to completely close the eyelids and obliterate the ordinary expression of the features. The inflamed surface was dark or purplish red, and both cheeks and eyelids were covered with a close aggregation of vessicles filled with a dark brown fluid. The extremities were cool; pulse small, weak, and 120 per minute; tongue partially covered with a reddish brown fur; lips dry; abdomen moderately tympanitic, and bowels loose. The patient was very restless, and complained of great exhaustion. He was directed to take tinct. ferri. murias. 20 gtts., every three hours, and a powder containing pulv. opii., 1½ grs., and sulph. quinine, 2 grs., between; also as much beef-tea and sweet-milk as his stomach would bear.

After pursuing this treatment thirty-six hours, there was no improvement in any of his symptoms. Dark brown liquid evacuations from the bowels continued to occur every three or four hours; the mind was somewhat wandering; the local erysipelatous inflammation remained stationary in its boundaries, but the vesicles had dried into *black* scabs, with evident ulcerations beneath them; while on the lower part of the right side of the face and the whole temporal and masseter region of the left, the skin had assumed that dark brown color, plainly threatening gangrene.

Regarding the case as one of great malignancy, from actual poisoning or degeneration of the blood, it was determined to try the effects of the *sulphites*. Accordingly the tincture of iron and quinine were both omitted, and one drachm of the *sulphite* of lime directed to be given every two hours.

To restrain the typhoid looseness of the bowels, a teaspoonful of the following emulsion was given between the doses of the sulphite of lime, viz.:—

℞. Ol. terebinth,....................................ʒii.

Tinct. opii.,.......................................ʒii.

Pulv. g. Arabic,.................................ʒiii.

Sachar. alba.......................................ʒiii.

Rub together thoroughly and add

Aqua mentha........................................℥ii. Mix.

Beef-tea and milk-porridge were continued for nourishment. At the end of twenty-four hours, after commencing this treatment, the only change observable in the patient was, a slower pulse, less mental wandering, and less frequent passages from the bowels. The same treatment was continued.

At the end of the second day, the pulse was 100 per minute, soft; skin generally cool; the bowels quiet; and the tumefaction of the face diminished. As the scabs began to separate from the cheeks, cicatrization was complete; but the skin and cellular tissue of the eyelids was extensively destroyed, leaving the left eye almost dissected from the socket. Over the places, on each side of the face, where gangrene was threatened, the skin had retained its vitality, but the cellular tissue beneath was evidently suppurating. The sulphite of lime was still continued every three hours, but the emulsion of turpentine and and tinct. opii. was continued only three times a day; same nourishment as before. The open ulcers around the eyes were washed with a weak solution of chloride of zinc, once a day. In two days more, making four days after the sulphite of lime was commenced, all the unfavorable constitutional symptoms had disappeared; but extensive abscesses had formed over the right side of the lower jaw and over the left temporal and masseter regions. These were freely opened, and the sulphite of lime continued in doses of one drachm four times a day. But as the diarrhœa had entirely ceased, the emulsion was discontinued; same nourishment continued. The patient continued steadily to improve for three days longer. The ulcers around the eyes were granulating healthily, and the abscesses on the face had nearly ceased to discharge, when another large abscess was found to exist under the scalp, directly on the vertex of the head. This was opened by a free incision, and a large amount of thin pus escaped, with some tough shreds of cellular tissue.

The patient was now attacked with catarrhal symptoms and pretty severe bronchial cough, for which, the following anodyne expectorant was administered, viz.:—

℞. Comp. honey of squills, &c., ........................℥j.
Tinct. bloodroot, ..........................................℥ss.
Camph. tinct. opii.,......................................℥jss.

mix, and give a teaspoonful every four hours; and continue one drachm of sulphite of lime morning and evening. In a few days the catharrhal symptoms subsided, and the patient passed on to complete recovery. But the contraction of the cicatrix in the left lower eyelid was sufficient to cause a moderate eversion, and make it difficult for the patient to close the eyelids fully.

CASE II.—Mrs. H., a native of Ireland, aged about 35 years, was admitted into the female ward of the Mercy Hospital, April

6th, 1863. She had been sick four or five days, and had taken some cathartic medicine.

At the time of her admission, her face, forehead, ears, and mastoid spaces were uniformly swollen of a dark purplish red color, on which were several vessications filled with dark serum. The surface of the face and trunk was hot and dry, but the extremities were cool. The mouth and tongue were dry; the pulse 118 per minute, soft and weak; bowels quiet for the last twenty-four hours; urine high-colored and scanty; slight subsultus, and mental faculties dull.

The treatment directed for this case was, one drachm of the *sulphite of lime* every three hours, and twenty drops of the tinct. ferri. murias, between, and the same nourishment as in the preceeding case. A manifest improvement had taken place in the general condition of the patient at the end of the first twenty-four hours,—the pulse was reduced to 110 per minute; the temperature of the extremities improved; and the mental faculties more active; although the erysipelatous inflammation had extended its boundaries over a greater portion of the scalp. The same treatment, medical and hygienic, was continued. At the end of the third day, the inflammation had entirely ceased to extend over more surface, with some abatement of the swelling in the face, and a still further improvement of the general symptoms. The same treatment was continued, except that the interval between the doses of medicine was lengthened, first to four, and, subsequently, to six hours. In one week, from the time of admission to the hospital, convalescence was fully established; and without either ulceration in the skin or suppuration in the sub-cutaneous cellular tissue.

Case III.—Mrs. C., a native of Ireland, aged about 38 years, was admitted into the same ward as the second case, April 10th, 1863. Five days previously she had had a miscarriage at the fourth month of pregnancy, which, so far as we could learn, had been attended by no unusual circumstances. On the third day after the miscarriage she was seized with a chill, pain in the back and hypogastric region, and fever. During the succeeding two or three days, we have no detailed account of her symptoms. But at the time of her admission into the hospital, her pulse was 130 per minute, very small and weak; her extremities cool; skin, generally, leaden color; countenance dull and relaxed; mind dull and sometimes wandering; abdomen distended, tympanitic, with acute tenderness over the whole lower half, and constant pain both in the lower part of the abdomen and in the lumbar and sacral regions. The evacuations from the bowels were frequent, dark brown, and thin. She was restless, and complained of inability to sleep.

From the small and frequent pulse, general prostration, and local pain and tenderness, it was evident that severe uterine phlebitis existed, with great danger of a fatal depression of the vital properties and degeneration of the blood. Hence, we directed the patient to take one drachm of the *sulphite* of lime every three hours, alternated with a powder containing pulv. opii., 1½ grs., sulph. quinine, 2 grs., and pulv. g. camphor, 3 grs., with milk-porridge for nourishment.

At the end of the first twenty-four hours the only changes observable in the condition of the patient were, less restlessness, pulse 120 per minute, less pain and tenderness in the lower part of the abdomen, and less frequent intestinal discharges.

The same treatment was continued. At the end of the second day the pulse was 112 per minute, but still, small, and weak; expression of countenance better; abdomen less tympanitic, and tender; and the bowels quiet. There was also a slight lochial discharge which had been previously suppressed. The *sulphite* of lime was continued as before, but the powders of opium, quinine, and camphor were given only three times a day. For nourishment, milk, milk-porridge, and animal broth.

At the end of the third day all symptoms of active uterine or phlebitic inflammation had disappeared, the diarrhœa had entirely ceased; and the patient expressed herself as, feeling almost well, except a sensation of nausea and occasional efforts to vomit, apparently, from the secondary effects of opium. The *sulphite* of lime was now continued only three times a day, and the powders of opium, &c., entirely omitted. To allay the irritability of the stomach, and exert a moderately tonic influence, the following mixture was directed, in doses of a teaspoonful every four hours, viz.:—

℞. Aromat. sulph. acid,..............................ʒiss.

Sulph. magnesia,..........................................℥ii.

Tinct. opii.,....................................................ʒii.

Aqua,.................. ,................................℥ii. Mix.

From this time the patient continued steadily to improve; and in two weeks, from the time of her admission, she was able to leave her bed. In commenting on these cases from time to time, to the class of students in attendance at the hospital, it was not claimed that they were sufficient to establish the efficacy of the *sulphite* of lime in the treatment of diseases dependent on blood-poisoning and degeneration. But they do show that the remedy may be given in large doses with perfect safety; and, taken in connection with the cases related by Dr. Fisher, in another part of this Journal, it is rendered highly probable that the *sulphites* will be found exceedingly valuable in the treatment of a whole class of severe and often fatal diseases.

